# A high-velocity star recently ejected by an intermediate-mass black hole in M15

**DOI:** 10.1093/nsr/nwae347

**Published:** 2024-10-03

**Authors:** Yang Huang, Qingzheng Li, Jifeng Liu, Xiaobo Dong, Huawei Zhang, Youjun Lu, Cuihua Du

**Affiliations:** School of Astronomy and Space Science, University of Chinese Academy of Sciences, Beijing 100049, China; National Astronomical Observatories, Chinese Academy of Sciences, Beijing 100012, China; Yunnan Observatories, Chinese Academy of Sciences, Kunming 650011, China; National Astronomical Observatories, Chinese Academy of Sciences, Beijing 100012, China; New Cornerstone Science Laboratory, National Astronomical Observatories, Chinese Academy of Sciences, Beijing 100012, China; Institute for Frontiers in Astronomy and Astrophysics, Beijing Normal University, Beijing 102206, China; Yunnan Observatories, Chinese Academy of Sciences, Kunming 650011, China; Department of Astronomy, School of Physics, Peking University, Beijing 100871, China; Kavli Institute for Astronomy and Astrophysics, Peking University, Beijing 100871, China; School of Astronomy and Space Science, University of Chinese Academy of Sciences, Beijing 100049, China; National Astronomical Observatories, Chinese Academy of Sciences, Beijing 100012, China; School of Astronomy and Space Science, University of Chinese Academy of Sciences, Beijing 100049, China

**Keywords:** intermediate-mass black hole, Hills mechanism, hypervelocity star, globular cluster, large-scale survey

## Abstract

The existence of intermediate-mass black holes (IMBHs) is crucial for understanding various astrophysical phenomena, yet their existence remains elusive, except for the LIGO-Virgo detection. We report the discovery of a high-velocity star J0731+3717, whose backward trajectory about 21 Myr ago intersected that of globular cluster M15 within the cluster tidal radius. Both its metallicity [Fe/H] and alpha-to-iron abundance ratio [$\alpha$/Fe] are consistent with those of M15. Furthermore, its location falls right on the fiducial sequence of cluster M15 on the color-absolute magnitude diagram, suggesting similar ages. These findings support the notion that J0731+3717 was originally associated with M15 at a confidence level of ‘seven nines’. We find that such a high-velocity star ($V_{\rm ej} = 548^{+6}_{-5}$ km s$^{-1}$) was most likely tidally ejected from as close as one astronomical unit to the center of M15, confirming an IMBH ($\ge 100 M_{\odot }$ with a credibility of 98%) as the exclusive nature of the central unseen mass proposed previously.

## Teaser

This paper reports the discovery of a high-velocity star J07 ejected from globular cluster M15 approximately 21 million years ago, providing strong evidence for the presence of an IMBH constrained to within a few AU of the central region of M15.

## INTRODUCTION

Intermediate-mass black holes (IMBHs) in the mass range of $10^2$–$10^5$ solar masses ($M_{\odot }$) may fill the gap between BHs formed as stellar remnants and supermassive BHs (SMBHs) found in the centers of galaxies [1]. Except for LIGO-Virgo detection [2,3], their existence, however, is still uncertain despite extensive search efforts. Discovering IMBHs and characterizing their mass functions in this range are therefore of great interest for many reasons [1].

Globular clusters (GCs), dense and massive dynamical systems, have long been considered promising places to harbor IMBHs. Both theory and numerical simulations suggest that IMBHs can form either through the repeated mergers of stellar black holes (‘slow mode’) [4], remnants of massive stars that sink to the center, or through the explosion of a very massive star resulting from runaway mergers of stars during an early phase of cluster core collapse (‘fast mode’) [5]. The ‘slow mode’ is a competitive scenario to explain the LIGO-Virgo-detected IMBHs [2,3] that arise from mergers of binary BHs.

Extensive efforts have indeed found a large central unseen mass in some globular clusters using velocity dispersion [6,7] or pulsar timing measurements [8], but such a mass can be either an IMBH or a cluster of stellar remnants within a few thousand astronomical units [9–11]. To exclude the latter possibility, we need to limit the central mass to a much smaller volume. One approach is to search for hyper/high-velocity stars ejected from globular clusters. If they exist, they are largely linked to the tidal interaction between an IMBH and a binary system for a close encounter, typically within one astronomical unit (AU).

## RESULTS AND DISCUSSION

### A high-velocity star ejected from globular cluster M15

To discover high-velocity stars ejected from globular clusters, backward orbital integrations are carried out for 934 high-velocity ($V_{\rm GSR} \ge 400$ km s$^{-1}$) halo stars in a searching volume of 5 kpc from the Sun [12] and 145 Galactic globular clusters [13–15]. Both the trajectories of the stars and globular clusters are traced back 250 Myr (in about one orbital period at solar position) using a common-adopted model of steady-state Galactic potential [16]. The closest distance for each high-velocity star and globular cluster pair is calculated from their backward trajectories. Amongst the hundred thousand pairs, only J0731+3717 has a closest distance smaller than the tidal radius of M15, making it a rare candidate for a cluster-ejected high-velocity star (see Sections A–F within the online supplementary material (SM) for more technical details).

**Figure 1.**
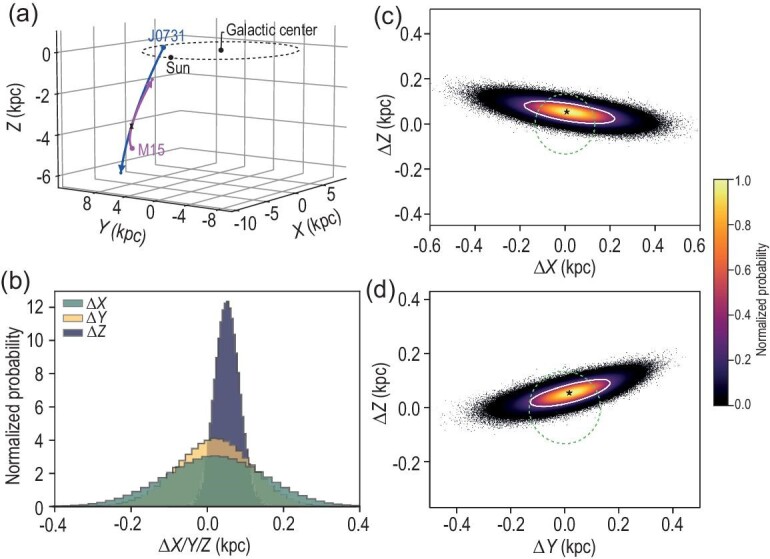
Backward orbital analysis of J0731+3717 and M15. (a) Three-dimensional representation of the backward orbits of J0731+3717 and the globular cluster M15. The blue and magenta lines with arrows mark the backward orbits of J0731+3717 and M15, respectively. The triangle and inverted triangle mark the encounter positions, which occurred 21 Myr ago, for M15 and J0731+3717. The black dots represent the locations of the Galactic center and the Sun, as labeled. The solar circle ($R = 8.178$ kpc) is shown with a dashed black line. (b) The distributions of the closest distance between J0731 and the center of M15 along $X$ (green), $Y$ (yellow) and $Z$ (purple) at encounter, estimated with one million MC trajectory simulations (see SM-B). (c, d) Density of the relative positions (J0731+3717 with respect to M15) at encounter, again estimated with one million MC trajectory calculations, in the (c) $\Delta X$-$\Delta Z$ and (d) $\Delta Y$-$\Delta Z$ planes. The normalized number density is color coded, as shown by the right color bar. The white contours mark the $1\sigma$ confidence region. The green dashed circle represents the size of the tidal radius of M15. The black star represents the relative location at closest distance derived directly using the observational parameters listed in Table 1.

Table 1 summarizes the information on J0731+3717 and M15. J0731+3717 is a high-velocity star with $V_{\rm GSR} = 419_{-6}^{+6}$ km s$^{-1}$ at a heliocentric distance of $1.295 \pm 0.013$ kpc. M15 is a 12.5-billion-year-old globular cluster [17] located in the constellation Pegasus with a heliocentric distance of $10.71 \pm 0.10$ kpc [14] and a mass of $5 \times 10^5\, M_{\odot }$ [18]. M15 is believed to host an IMBH of 1700–3200 $M_{\odot }$ based on velocity dispersion measurements [6,19], albeit with debate [9,20,21], especially the $3\sigma$ upper limit of 980 M$_{\odot }$ placed by the non-detection from ultra-deep radio observations at the cluster core [22]. While J0731+3717 is currently 11.5 kpc away from M15, their backward trajectories intersected with each other 21 Myr ago with a relative velocity of $548_{-5}^{+6}$ km s$^{-1}$ and a closest distance of 58 pc (Fig. 1a), smaller than the cluster tidal radius of 132 pc. The intersection locations and their uncertainties in three-dimensional space are estimated with one million Monte Carlo (MC) trajectory simulations to be $\Delta X = 20_{-135}^{+122}$ pc, $\Delta Y = 19_{-104}^{+95}$ pc and $\Delta Z = 51_{-32}^{+32}$ pc, as shown in Fig. 1b&d.

**Table 1.** The measured parameters of J0731+3717 and M15.

Parameter	J0731+3717	M15	Units
RA (J2000)	07:31:27.26	21:29:58.33	hh:mm:ss.ss
Dec (J2000)	$+$ 37:17:04.3	$+$ 12:10:01.2	dd:mm:ss.s
*Gaia* DR3 source_id	898707303799931392	N/A	N/A
*Gaia* DR3 proper motion $\mu _{\alpha }\rm {cos}\, \delta$	$47.963 \pm 0.041$	$-0.659 \pm 0.024$	mas yr$^{-1}$
*Gaia* DR3 proper motion $\mu _{\delta }$	$ -83.666 \pm 0.035$	$-3.803 \pm 0.024$	mas yr$^{-1}$
*Gaia* DR3 parallax	$0.733 \pm 0.042$	$0.097 \pm 0.010$	mas
*Gaia* DR3 $G$-band magnitude	$15.814 \pm 0.003$	N/A	mag
*Gaia* DR3 $G_{\rm BP} - G_{\rm RP}$	$0.765 \pm 0.007$	N/A	mag
SDSS $u$-band magnitude	$17.095 \pm 0.008$	N/A	mag
SDSS $g$-band magnitude	$16.194 \pm 0.004$	N/A	mag
SDSS $r$-band magnitude	$15.832 \pm 0.004$	N/A	mag
SDSS $i$-band magnitude	$15.693 \pm 0.004$	N/A	mag
SDSS $z$-band magnitude	$15.643 \pm 0.007$	N/A	mag
Distance	$1295.2 \pm 13.1$	$10709.0 \pm 95.5$	pc
$E (B - V)$	0.047	0.08	mag
Heliocentric radial velocities HRV	$196.68 \pm 6.97$	$-107.0 \pm 0.2$	km s$^{-1}$
Effective temperature $T_{\rm eff}$	$6062.0 \pm 157.0$	N/A	K
Surface gravity log $g$	$4.02 \pm 0.29$	N/A	dex
Metallicity [Fe/H]	$-2.23 \pm 0.13$	$-2.33 \pm 0.02\ \text{(stat.)} \pm 0.10$ (syst.)	dex
$\alpha$ -element-to-iron ratio [$\alpha$/Fe]	$+0.24 \pm 0.07$	$0.24 \pm 0.03$	dex
Total velocity $V_{\rm GSR}$	$418.71^{+6.54}_{-5.95}$	$111.85^{+1.48}_{-1.35}$	km s$^{-1}$
Age	$13.00^{+1.75}_{-2.00}$	$12.5 $	Gyr
Mass	$0.69^{+0.02}_{-0.01}$	$4.94 \times 10^{5}$	$M_\odot$

**Figure 2.**
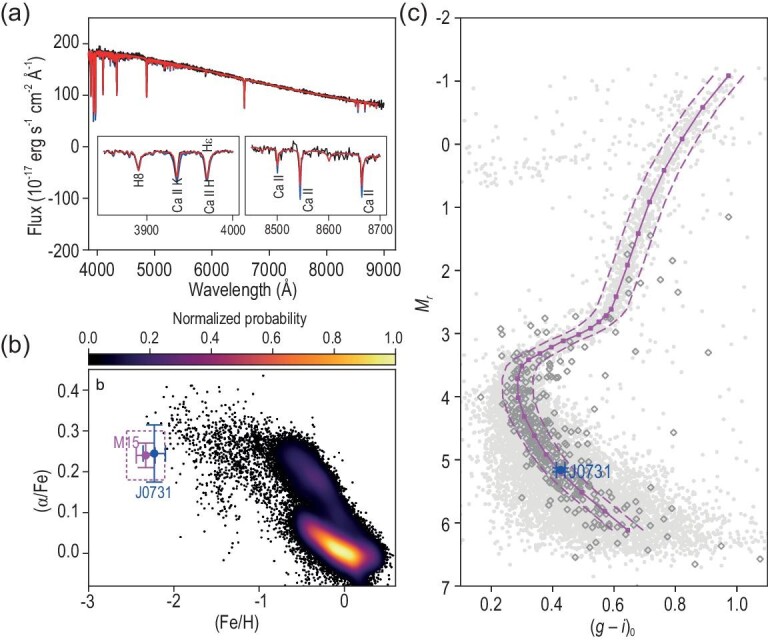
Optical spectrum, chemical properties and color-absolute magnitude diagram of J0731+3717 and M15. (a) Optical spectrum (in black) of J0731+3717 from the SEGUE survey. Two synthetical spectra (degraded to SEGUE spectral resolution) are plotted on top of each other for comparison (see SM-E). The red spectrum has stellar parameters $T_{\rm eff} = 6100$ K, log $g = 4.0$, $\text{[Fe/H]}=-2.0$ and $\text{[$\alpha $/Fe]}=+0.20$, similar to those of J0731+3717, while the blue spectrum is 0.5 dex richer in metallicity (i.e. $\text{[Fe/H]}=-1.5$) with the other parameters unchanged. The insets show enlarged views of the Ca ii H ($\lambda$3968) and K ($\lambda$3933) lines, and the Ca ii triplet lines at $\lambda \lambda$8498, 8542, 8662. (b) [Fe/H]–[$\alpha$/Fe] diagram for J0731+3717 (blue dot) and globular cluster M15 (magenta dot). The dashed magenta box marks the region within two times the measurement uncertainties of [Fe/H] and [$\alpha$/Fe] for M15. For comparison purposes, the background shows the density of APOGEE-targeted stars with reliable determinations of [Fe/H] and [$\alpha$/Fe] (see SM-H). (c) Plot of $M_r$ versus $(g - i)_0$ for globular cluster M15 and J0731+3717. The background gray dots are photometric observations of M15 from the Galactic globular and open clusters study based on the Sloan Digital Sky Survey by An *et al.* [26], by adopting a cluster distance of 10.71 kpc and an $E (B-V)$ value of 0.08 (Table 1). The magenta squares denote the cluster fiducial sequence derived from the background gray dots by An *et al.* [26]. The dashed magenta lines are shifted from the fiducial sequence by $\pm 0.05$ mag in $(g-i)_0$. The diamonds are field halo stars from the existing spectroscopic surveys with chemical fingerprints (defined by the magenta box shown in panel (b)) similar to M15 (see SM-H).

In addition to the orbital connection, J0731+3717 exhibits rare chemical fingerprints consistent with those of M15. The SEGUE spectrum [23] clearly shows that J0731+3717 is a very metal-poor late F-type star, as plotted in Fig. 2a, with effective temperature $T_{\rm eff} = 6062 \pm 157$ K, metallicity [Fe/H] = $-2.23 \pm 0.13$ and alpha-to-iron abundance ratio $\text{[$\alpha $/Fe]} =+0.24 \pm 0.07$ (see SM-E). The old globular cluster M15 has well measured ${\rm [Fe/H]}=-2.33 \pm 0.10$ and $\text{[$\alpha $/Fe]}=+0.24 \pm 0.03$ [24,25]. Their chemical parameters are consistent with each other within errors, and both are located in a region with very few stars in the [Fe/H]-[$\alpha$/Fe] plane. Only $7.5 \times 10^{-3}$ of all halo stars within the 5-kpc searching volume with reliable abundances are located in such a region, as shown in Fig. 2b. The rare chemical similarity implies that J0731+3717 was originally associated with the cluster, in line with the suggestion of orbital analysis.

The association of J0731+3717 with M15 can be further supported by their similar ages as derived from isochrone fitting. Broadband photometric measurements for M15 and J0731+3717 are taken from the Galactic globular and open clusters study based on the Sloan Digital Sky Survey (SDSS) [26] and SDSS DR12 [27], respectively. Both their absolute magnitudes and colors are corrected for extinction values along their separate lines of sight. As shown in Fig. 2c, J0731+3717 falls right on the cluster fiducial sequence of M15; subsequently, its isochrone age as estimated from the Bayesian approach is $13.00^{+1.75}_{-2.00}$ Gyr, almost identical to the age obtained for M15 (see SM-F & G). In comparison, 37% of the field halo stars with chemical abundances similar to M15 (defined by the box in Fig. 2b) actually deviate from its fiducial sequence by more than 0.05 mag (the maximal error) in color direction, implying significantly different ages.

It is extremely unlikely for the association of J0731+3717 and M15 to be by pure chance, given the probability for random association and chemical and age similarities. To quantitatively determine the probability of random high-velocity halo stars encountering M15, we ran MC simulations to generate about one million high-velocity halo stars with $V_{\rm GSR} \ge 400$ km s$^{-1}$ in such a searching volume (see SM-H). Only 12 high-velocity stars have had close encounters with M15 within its tidal radius in the past 250 Myr. This means that the probability of an unphysical orbital encounter between J0731+3717 and M15 is $1.2 \times 10^{-5}$. Considering the orbital link, together with the chemical and age similarities, one high-velocity halo star in our searching volume is coincidentally linked to M15 by a pure chance of only $5.89 \times 10^{-8}$. In other words, J0731+3717 is a true former member of M15 at a confidence level of ‘seven nines’.

### Interpretation as a Hills ejection via an IMBH

**Figure 3.**
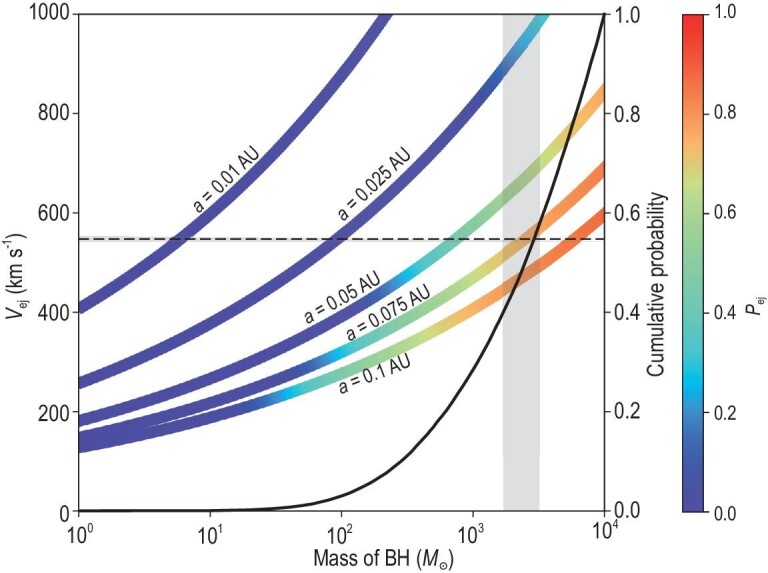
Ejection velocities predicted by the Hills mechanism. The lines represent the most probable ejection velocities, calculated using Equations S7 and S8 within the SM-I, as a function of the black hole mass under different binary separations ranging from 0.01 to 0.1 AU (from left to right). In the calculation, the binary is assumed to contain two J0731+3717-like stars, each with a mass of $0.69 M_{\odot }$. The color of the lines indicates the ejection probability (see the color bar shown on the right), derived using Equation S9 within the SM-I. Here the closest distance that the binary can approach the black hole is set to 0.5 AU. The horizontal dashed line with a shaded $1\sigma$ uncertainty indicates the reported ejection velocity ($548^{+6}_{-5}$ km s$^{-1}$) of J0731+3717. The vertical shaded region represents the mass of the black hole (1700-3200 $M_{\odot }$) of M15 dynamically derived from velocity dispersion measurements [6,19]. The black line represents the cumulative probability of the ejection of 0731+3717-like stars at different black hole masses, calculated through Monte Carlo simulations (see SM-I).

An energetic ejection mechanism is required to kick-off J0731+3717 from M15 with an ejection velocity up to $548^{+6}_{-5}$ km s$^{-1}$; in comparison, the cluster central escape velocity is only 62 km s$^{-1}$ (the escape velocity at the cluster half-mass radius is 27 km s$^{-1}$; see [28]). The Hills mechanism [29] invokes three-body exchange interactions between stellar binary and super-massive BHs to eject hypervelocity stars from the Galactic center; such a mechanism can naturally eject high-velocity stars from IMBHs in the center of globular clusters. If this mechanism is at work in this case, the ejection velocity would constrain the mass of the central BH in M15 to be 726 $M_{\odot }$ for a binary separation $a = 0.05$ AU and 5804 $M_{\odot }$ for $a = 0.10$ AU. A stellar-mass BH smaller than $100\ M_{\odot }$ can also eject a star up to around 550 km s$^{-1}$ if $a \le 0.025$ AU, but the probability is only a few percent based on our MC simulations of two hundred million Hills ejections (see SM-I and Fig. 3). Thus, the ejection of J0731+3717 from M15 requires an IMBH ($\ge 100\ M_{\odot }$ with a credibility of 98%) at the center of the cluster; the encounters mostly occurred (93%) within 2 AU from the cluster center. This confirms that the previously claimed aggregated mass of more than a few thousand solar masses [6,19,22] is indeed an IMBH rather than a cluster of stellar remnants [20,21].

### Excluding alternative explanations

A competing mechanism to eject high-velocity stars (even up to 2000 km s$^{-1}$) in a globular cluster is the single star–binary interaction involving compact objects. Observations reveal the presence of a binary neutron star, possibly a remnant from these encounters in M15 [30]. However, the ejection rate, as derived by a recent comprehensive Monte Carlo $N$-body simulation [31], for producing J0731+3717-like high-velocity stars remains remarkably low, $\sim\! 4 \times 10^{-8}$ yr$^{-1}$ in the present day,  which is three orders of magnitude lower than that of the aforementioned IMBH-binary encounters (see SM-J). The same simulation shows that no J0731+3717-like high-velocity stars with $V_{\rm GSR} \ge 400$ km $^{-1}$ were kicked from M15 through this channel in the past 10 Gyr (let alone the past 250 Myr).

A high ejection velocity can be obtained through several alternative scenarios. First, a star can be ejected through a normal single star–binary interaction [32] without compact objects involved or exchange collision with a massive star [33] (similar to the Hills mechanism but for massive stars), but the typical ejection velocity is within 200 km s$^{-1}$. To eject a J0731+3717-like star with an ejection velocity above 550 km s$^{-1}$, interaction between a very massive star (50–100 $M_{\odot }$) and a hard massive binary is required [33], which cannot have recently occurred in such an old cluster like M15. Second, a star can be ejected by the core-collapse supernova explosion of its former massive companion in a binary scenario [34]. However, this is unlikely to have recently occurred in M15 in light of its old age; moreover, the maximum kick velocity by supernova would be largely within 300-400 km s$^{-1}$ [35]. At present, only type-Ia supernovae from a white dwarf plus helium star (hot subdwarf) or the dynamically driven double-degenerate double-detonation [36,37] channels can eject the surviving helium star or white dwarf with velocity even up to 1000 km s$^{-1}$ like US 708 [38] or like D6-1 to D6-3 [37]. One possible such ejection associated with the GC was recently reported in the nearby galaxy NGC 5353 [39]. The late F-type nature of J0731+3717 certainly rules out the possibility of a fast helium star or white dwarf ejected by a type-Ia supernova explosion. Third, a star can be stripped from the cluster when the latter experiences tidal shock from interactions with giant molecular clouds [40], the Galactic disk [41], the spiral arm [42] or perigalactic passages [41]. Apparently, J0731+3717 is not such a case since the ejection position (at $-3.6$ kpc below the Galactic disk plane; see Fig. 1a) is far away from the Galactic disk, known giant molecular clouds or the last pericenter of M15. In summary, the above alternative ejection mechanisms are not viable to kick off J0731+3717 from M15.

## CONCLUSION

Our discovery of J0731+3717 ejected by an IMBH in M15 thus proves that the existence of IMBHs can be disclosed by high-velocity stars ejected from clusters via the Hills mechanism, unambiguously as compared to previous velocity dispersion measurements [6,9,20,21]. Such a method can be applied to find more cases like J0731+3717 in our Galaxy. Simulations of the 145 globular clusters in the past 14 Gyr led to around 500 J0731+3717-like high-velocity stars ejected into the current 5-kpc searching volume (see SM-J), although only 50 of them were ejected in the past 250 Myr that can be traced back to their cluster origin under current measurement uncertainties. With the increasing power of ongoing *Gaia* and large-scale spectroscopic surveys, we expect to discover dozens of cases within the 5-kpc volume and ten times more within a 10-kpc volume, which should shed light on understanding the evolutionary path from stellar-mass BHs to SMBHs.

## METHODS

Detailed methods are available in the online supplementary material.

## Supplementary Material

nwae347_Supplemental_File

## Data Availability

All data used in this study are publicly available. This work made use of data from the European Space Agency (ESA) mission Gaia (https://www.cosmos.esa.int/gaia), processed by the Gaia Data Processing and Analysis Consortium (DPAC; https://www.cosmos.esa.int/web/gaia/dpac/consortium). The stellar parameters of J0731+3717 are available from the SDSS-II/III SEGUE archive at https://data.sdss.org/sas/dr12/sdss/sspp/ssppOut-dr12.fits. The SDSS photometric and spectroscopic data can be found at http://skyserver.sdss.org/dr12/en/tools/search/radial.aspx. The photometric catalog of M15 is available from http://das.sdss.org/va/osuPhot/v1_0/. The stellar isochrones can be found at http://stellar.dartmouth.edu/models. The data supporting the plots in this paper and other findings of this study are available from the corresponding authors upon reasonable request. We used standard data analysis tools in Python environments. Specifically, the orbital analysis was carried out with Python package Gala and galpy, which are publicly available at http://gala.adrian.pw/en/v1.6.1/getting_started.html and www.galpy.org.
