# Supplementary material for: A high-velocity star recently ejected by an intermediate-mass black hole in M15
Source: Natl Sci Rev. 2024 Oct 3;12(2):nwae347. doi: 10.1093/nsr/nwae347 (PMC11809261; doi:10.1093/nsr/nwae347)
Supplement: nwae347_Supplemental_File [file nwae347_supplemental_file.pdf]

Supplementary Materials for

A high-velocity star recently ejected from the globular cluster M15

Yang Huang[1,2,†,✉], Qingzheng Li[3†], Jifeng Liu[2,4,5,✉], Xiaobo Dong[3,✉], Huawei Zhang[6,7,✉], Youjun Lu[1,2], Cuihua Du[1]

[1]School of Astronomy and Space Science, University of Chinese Academy of Sciences, Beijing 100049, China;
[2]National Astronomical Observatories, Chinese Academy of Sciences, Beijing 100012, China;
[3]Yunnan Observatories, Chinese Academy of Sciences, Kunming 650011, China;
[4]New Cornerstone Science Laboratory, National Astronomical Observatories, Chinese Academy of Sciences, Beijing 100012, China;
[5]Institute for Frontiers in Astronomy and Astrophysics, Beijing Normal University, Beijing, 102206, China;
[6]Department of Astronomy, School of Physics, Peking University, Beijing 100871, China;
[7]Kavli Institute for Astronomy and Astrophysics, Peking University, Beijing 100871, China;
† These authors contributed equally;
✉ Corresponding authors: jfliu@nao.cas.cn; huangyang@ucas.ac.cn; xbdong@ynao.ac.cn; zhanghw@pku.edu.cn.

Supplementary Materials

A Coordinate systems.

We adopt two sets of coordinate systems: (1) a right-handed Cartesian coordinate system (X, Y, Z), with positive X direction pointing towards the Galactic centre from the Sun, Y pointing to the direction of Galactic rotation of the Sun, and Z in the direction of north Galactic pole; (2) a Galactocentric cylindrical system (R, ϕ, Z), with R the projected Galactocentric distance increasing radially outwards, ϕ the azimuthal angle towards the direction of the Galactic rotation, and Z the same as that in the Cartesian system. The Sun is set to (R_0, $Z_\odot$) = (8.178, 0.025) kpc (ref.[1,2]) and the circular velocity at the solar position is fixed to $V_c(R_0) = 220\,\mathrm{km\,s^{-1}}$ (ref.[3]). The solar motions with respect to the local standard of rest are set to $(U_\odot, V_\odot, W_\odot) = (7.01, 10.13, 4.95)\,\mathrm{km\,s^{-1}}$ (ref.[4]).

B Systematic search.

By combing datasets of the RAVE DR5 (ref.[5]), SDSS DR12 (ref.[6]), LAMOST DR8 (ref.[7]), APOGEE DR16 (ref.[8]), GALAH DR2[9], and *Gaia* DR3 (ref.[10]), a large sample of 943 high-velocity halo stars with total velocity $V_{\rm GSR} \geq 400\,\mathrm{km\,s^{-1}}$ is constructed[11]. The distances of those stars are first estimated from the parallax measurements provided by *Gaia* DR3 using a Bayesian approach[11]. The distance estimates are further improved by adding constraints from SDSS optical colors and chemical information (i.e., metallicity and [α/Fe] from spectroscopic surveys) into the Bayesian analysis, which will be introduced in the latter section. Their 3D velocities are then calculated from the measured distances, proper motions again from *Gaia* DR3 and heliocentric radial velocities (HRV) provided by the aforementioned spectroscopic surveys, except for the SDSS survey, whose HRV is carefully re-determined by the LAMOST stellar parameter pipeline at Peking University (LSP3; ref.[12]) utilizing specific template of similar atmospheric parameters for each star (see technique details in the latter section). Moreover, they have metallicities and alpha-to-iron abundance ratios well measured from their spectra. We perform a systematic search for high-velocity stars ejected from globular clusters. To do so, backward orbital integrations are carried for those 943 high-velocity halo stars and 145 Galactic globular clusters with well measured metallicities, distances, proper motions and HRVs (refs.[21,22,23]). For the orbital analysis, we adopt the python package `Gala`[13] with the Galactic potential setting to `MilkyWayPotential`[3], which contains four components: a nucleus, a bulge, a disk, and a dark matter halo. In this study, we only concern recent ejections during the past 250 Myr since the dynamical effects (e.g., the dynamical friction[14]), that globular cluster experienced, are hard to be precisely considered in the backward orbital calculations. The trajectories are therefore traced back in time of 250 Myr with a time resolution of 1 kyr. To discover cluster ejected high-velocity candidates, the trajectories of each high-velocity star and globular clusters are carefully investigated by sorting the ratios between the closest orbital distances $d_{\rm min}$ and the tidal radii $r_{\rm t}$ of the clusters which can be found at `https://people.smp.uq.edu.au/HolgerBaumgardt/globular/parameter.html`. The final candidates are required to have the ratios ($d_{\rm min}/r_{\rm t}$) smaller than one and metallicity differences between the high-velocity stars and the clusters smaller than 0.2 dex. During this search, we identified J0731+3717 (the only one of 135,430 trajectory pairs), a high-velocity star has a close encounter with M15 within its tidal radius about 21 Myr ago. The closest orbital distance is $58.0^{+117.4}_{-58.0}$ pc, which is smaller than the tidal radius ($r_{\rm t} = 132.1$ pc) of M15 (see Extended Data Table 1 for top 5 clusters sorted by ratios of $d_{\rm min}/r_{\rm t}$). The encounter occurred where J0731+3717 is located at (X, Y, Z) = $(-5.91^{+0.13}_{-0.14}, 7.19^{+0.10}_{-0.10}, -3.61^{+0.04}_{-0.04})$ kpc with (V_X, V_Y, V_Z) = $(-205.16^{+6.07}_{-6.68}, -305.94^{+6.23}_{-6.85}, 171.19^{+3.06}_{-3.37})\,\mathrm{km\,s^{-1}}$, and M15 is located at (X, Y, Z) = $(-5.93^{+0.03}_{-0.03}, 7.17^{+0.09}_{-0.09}, -3.66^{+0.03}_{-0.03})$ kpc with (V_X, V_Y, V_Z) = $(56.18^{+2.15}_{-1.95}, 103.13^{+1.26}_{-1.26}, -82.10^{+1.38}_{-1.30})\,\mathrm{km\,s^{-1}}$. At the closest approach, J0731+3717 had a relative velocity of $541.10^{+5.96}_{-5.42}\,\mathrm{km\,s^{-1}}$ with respect to M15 (see Extended Data Figure 1). The upper and lower uncertainties of these reported parameters from our orbital analysis come from 16 and 84 per cent percentiles of the probability distribution function (PDF) yielded by one million MC trajectory calculations. During the calculations, the astrometric parameters (except the parallax/distance) of J0731+3717 are assumed to be multivariate Gaussian distributions with the correlation coefficients (taken from *Gaia* DR3) considered. The distance of J0731+3717 is assumed to follow the posterior PDF derived from our Bayesian analysis. The HRV of J0731+3717 is uncorrelated to astometric parameters and thus assumed normally distributed. For M15, the uncertainties in positions, distance, proper motions and HRV are taken from refs[22,23]. and are all assumed to follow normal distributions.

We note that the last cross with the disk is happened 2.6 Myr ago with the intersect positions at $(X_p, Y_p) = (-9.1, 0.9)$ kpc, far away from the Galactic center. This result clearly rules out the possibility of ejecting J0737+3717 from the supermassive BH at the Galactic center. In addition to the above 250 Myr orbital integrations, the whole past 14 Gyr (the age of universe) backward orbits of J0731+3717 and globular clusters, as well as the best-known dwarf galaxies (parameters are adopted from ref.[15]), are investigated. No other orbital links are found. The whole backward orbits of J0731+3717 are also provided as an online supplementary data.

C Orbital deflections.

During the backward orbital integrations, the deflections caused by encounters with field stars are ignored. We thus here evaluate this effect on orbital analysis. The deflection angle is defined as[16]:

$$\theta_{\rm defl} = 2\tan^{-1}(b_{90}/b), \tag{S1}$$

where b is the distance of the close approach, and b_{90} is the $90°$ deflection radius that is given by:

$$b_{90} = \frac{G(m_1 + m_2)}{V_0^2}, \quad \text{(S2)}$$

where m_1 and m_2 are the masses of two stars having close meet, V_0 is the relative speed. By assuming two solar-like stars with $V_0 = 300\,\mathrm{km\,s^{-1}}$, the deflection angle is only about 0.039 arcsec for a 1 pc encounter. The Sun is expected to experience 1 pc encounter at a rate of 19.7 ± 2.2 Myr^{-1}[17]. During our orbital calculations with a total integration time of 250 Myr, the maximum deflection angle for solar-like stars at solar position is within 3.5 arcmin, which is much smaller than the tidal radius of M15 (42.4 arcmin for $r_\mathrm{t} = 132$ pc at 10.7 kpc). In our case, the deflection angles of these halo stars are even much smaller, given their much lower spatial number density. Specifically, for J0731+3717 with typical $V_0 = 420\,\mathrm{km\,s^{-1}}$, the deflection angle is smaller than 8.4 arcsec (0.44 pc at the distance of M15), even assuming a high encounter rate at solar position. This deflection angle is not only much smaller than the tidal radius of M15 or uncertainty of meet distance (see above section) but also significantly less than its half-light radius (one arcmin from ref.[21]). Thus, the orbital deflection has minor effects on our backward orbital analysis.

D Orbital analysis with alternative assumptions.

To show the robustness of our backward orbital reconstructions, we repeat the whole analysis by adopting alternative assumptions. In total, six tests are performed. In each test, only one assumption is changed and other parameters remain the same. First, we change the Galactic potential to `BovyMWPotential2014`[3] consisting of three components: a bulge, a disk, and a dark matter halo. In the second test, alternative values of solar motions with respect to the local standard of rest $(U_\odot, V_\odot, W_\odot) = (11.10, 12.24, 7.25)\,\mathrm{km\,s^{-1}}$ are adopted[18]. In the third test, we use the circular velocity at the solar position $V_c(R_0) = 234.04\,\mathrm{km\,s^{-1}}$ (ref.[19]). In the fourth test, the Galactocentric distance of the sun R_0 is changed to 8.34 kpc[20]. For the fifth test, M15 is modelled as a moving potential with a Plummer profile, calculated by the `MovingObjectPotential` function implemented in `galpy` (ref.[3]). The size and mass of M15 is taken from ref.[23]. Finally, we consider the effects from the potential of the Large Magellanic Cloud (LMC), by modelling it as a Plummer profile with a total mass of $1.38 \times 10^{11}\,\mathrm{M_\odot}$ and a scale radius of 17.14 kpc (ref.[24]). The sky positions, distances, proper motions and HRV of LMC are adopted from ref.[25]. The moving potential of LMC is again calculated by `MovingObjectPotential`. We also include the dynamical friction on the LMC from the Milky Way by the `ChandrasekharDynamicalFrictionForce` function of `galpy`. The closest encounter distances as found by the above tests are all within 60 pc (see Table S2), smaller than the tidal radius ($r_t = 132.1$ pc) of M15. The backward time of the meets are all around 21 Myr, in great consistency with our default result. The comprehensive tests show that the impact of alternative assumptions on the backward orbital analysis is negligible.

E SEGUE spectra and parameters of J0731+3717.

Two optical spectra of J0731+3717 have been obtained by the SEGUE survey[26] on 2000 November 29 and 2005 March 17, with a signal-to-noise-ratio (SNR) of 49.4 and 55.8, respectively. The spectra have covered the full optical range (3800-9200Å) with a resolving power of around 2000. Stellar parameters and heliocentric radial velocity (HRV) are derived from the observed spectra by the SEGUE Stellar Parameter Pipeline (SSPP)[27]. The typical precisions are 157 K, 0.29 dex, 0.13 dex, 0.07 dex and 5 km s^{-1} for effective temperature T_eff, surface gravity $\log g$, metallicity [Fe/H], alpha-to-iron abundance ratio [α/Fe], and heliocentric radial velocity (HRV), respectively[27,28]. Two groups of stellar parameters are derived by SSPP from the two visits and they are consistent with each other very well, showing the robustness of the pipeline. We here adopt the stellar parameters and HRV (Table 1) measured from the spectrum with higher SNR, i.e. the one observed on 2005 March 17. The spectrum is shown in Fig. 2a. Clearly, the SEGUE spectrum indicates that J0731+3717 is a real very metal-poor star with [Fe/H] down to -2 and even lower, directly shown by the comparisons with the synthetical spectra adopted from Göttingen spectral library[29] (see Figure 2b).

Given the metal-poor nature of J0731+3717, we re-estimate its HRV and uncertainty by using a specific metal-poor template. To do so, we adopted the LSP3 that is developed to derive HRVs from LAMOST/SEGUE-like low resolution spectra using cross-correlating technique with ELODIE library[30] as template (degraded to SEGUE resolution). We note the SEGUE spectrum (observed on 2005 March 17) of J0731+3717 was corrected for a systematic offset of $+7.3\,\mathrm{km\,s^{-1}}$ due to wavelength calibrations (ref.[31]; `https://www.sdss3.org/dr9/algorithms/wavelength.php`). For the template, we choose BD+023375 ($T_\mathrm{eff} = 5944$ K, $\log g = 3.97$ and [Fe/H] = -2.29; ref.[30]) star included in the ELODIE library, whose atmospheric parameters are very close to these of J0731+3717. We then run LSP3 to the SEGUE spectrum of J0731+3717 and find its HRV of $196.68 \pm 6.97\,\mathrm{km\,s^{-1}}$. The 1σ error is properly estimated using metal-poor stars with multiple observations in LAMOST. This uncertainty is slightly larger than the typical one ($5\,\mathrm{km\,s^{-1}}$) for normal metal-rich FGK stars but in great consistency with the independent check from metal-poor globular clusters (ref.[28]). As mentioned in previous systematic search section, the HRVs of other high-velocity stars from SDSS DR12 are also re-determined by LSP3 in the above manner.

F Mass, age and improved distance of J0731+3717.

We determine the mass, age and improved distance of J0731+3717 by the common used Bayesian approach. In this approach, the PDF of the age, mass and absolute magnitudes is expressed as:

$$f(\tau, m, M_\lambda) = NP(\tau, m)\mathcal{L}(\tau, m), \tag{S3}$$

where $\lambda = u, g, r, i, z$ and N is a normalization parameter that ensures $\iint f(\tau, m) d\tau dm = 1$. For $P(\tau, m)$, a uniform prior and a Salpeter initial mass function[32] are assumed for age and mass, respectively. The likelihood function $\mathcal{L}$ is obtained by:

$$\mathcal{L} = \prod_{i=1}^{n} \frac{1}{\sqrt{2\pi}\sigma_i} \times \exp(-\chi_i^2/2), \tag{S4}$$

where

$$\chi_i^2 = \left(\frac{O_i - M_i(\tau, m)}{\sigma_i}\right)^2. \tag{S5}$$

Here O_i represents the observational constraints from the intrinsic color $(g-i)_0$, absolute magnitudes M_λ and metallicity [Fe/H], M_i represents the model values from the stellar isochrones, taken from the Dartmouth Stellar Evolution Program (DSEP)[33], at a given τ and M. The total number of observed parameters is n, and σ_i is the uncertainty of the ith observed parameter. The intrinsic color $(g-i)_0$ of J0731+3717 is from SDSS photometry after correcting for the dust reddening. The five SDSS band absolute magnitudes are given by combination of SDSS photometry with reddening corrected and the distance of J0731+3717 is measured from *Gaia* parallax. Here the value of the reddening is taken from the SFD map[34] (after correction of 14% systematics[35]) and the extinction coefficients are adopted from ref.[35]. The metallicity [Fe/H] is derived from the SEGUE spectra. The uncertainties of these observational constraints (as listed in Table 1) are well considered in the estimation. For the DSEP isochrones, a constant value of $+0.20$ dex of [α/Fe], close to that measured for J0731+3717, is adopted.

The resulted PDFs yield a mass of $0.69^{+0.02}_{-0.01}\,\mathrm{M}_\odot$, an age of $13.00^{+1.75}_{-2.00}$ Gyr for J0731+3717 and a weighted mean distance modulus of 10.56 ± 0.02 (corresponding to $d = 1295.2 \pm 13.1$ pc) from the five SDSS bands. We note this method is also adopted to improve the distance estimate of other high-velocity stars in previous section of systematic search. In Figure S2, we compare J0731+3717 to the DSEP isochrones on the M_r–$(g-i)_0$ diagram to show the robustness of the resulted parameters. The derived age of J0731+3717 agrees very well with that determined for M15.

G Color-absolute magnitude diagram of M15.

We adopt the photometric measurements from the Sloan Digital Sky Survey Galactic globular and open clusters project[36] to construct the color-absolute magnitude diagram of M15. This project presents precise photometry for 17 Galactic globular clusters and open clusters by using the DAOPHOT/ALLFRAME procedure[37,38] that are developed for crowded fields. We select M15 member stars from the catalog yielded by the project using the following criteria: 1) stars are within 6 arcmin from the center of M15; 2) stars in gri-bands are required with DAOPHOT parameters: $|\mathrm{sharp}| < 1$ and $\chi < 1.5 + 4.5 \times 10^{-0.4(m-16.0)}$; 3) stars have photometric uncertainties smaller than 0.2 mag in gri-bands. In total, over ten thousand stars are left from the above cuts. By adopting a cluster distance of 10.71 kpc (ref.[23]) and a dust reddening of $E(B-V) = 0.08$ (ref.[39]), the color-absolute magnitude is constructed for M15 (see grey dots in Figure 2c). We further convert the cluster fiducial sequence (the locus of the number density peaks, taken from ref.[36]) on r versus $(g-i)$ plane to that on M_r versus $(g-i)_0$ plane, by adopting the cluster distance and reddening values. The results are shown in Figure 2c as the magenta squares.

H Confidence level of the ejection of J0731+3717 from M15.

We calculate the possibility that one high-velocity halo star (in the 5 kpc searching volume) is linked to M15 by pure chance. First, we generate 50 million stars randomly distributed in a local volume of $(5\mathrm{kpc})^3$ from the Sun (similar to the searching volume of high-velocity stars[11]) following velocity distributions of the local halo stars:

$$f(v_R, v_\phi, v_Z) = k \exp\left(-\frac{v_R^2}{2\sigma_R^2} - \frac{(v_\phi - \overline{v_\phi})^2}{2\sigma_\phi^2} - \frac{v_Z^2}{2\sigma_Z^2}\right), \tag{S6}$$

where $k = \frac{1}{(2\pi)^{3/2}\sigma_R\sigma_\phi\sigma_Z}$, $\overline{v_\phi} = +35.53\,\mathrm{km\,s^{-1}}$, $\sigma_R = 150.57\,\mathrm{km\,s^{-1}}$, $\sigma_\phi = 115.67\,\mathrm{km\,s^{-1}}$ and $\sigma_Z = 86.67\,\mathrm{km\,s^{-1}}$ (ref.[40]). In total, around one million stars (964,630) are found at the high-velocity tail with $V_{\mathrm{GSR}} \geq 400\,\mathrm{km\,s^{-1}}$. We thus perform backward orbital analysis for those high-velocity halo stars and the globular cluster M15. The integration settings are the same as those detailed in systematic search section. During the calculations, only 12 mock high-velocity halo stars have the chances to meet M15 within its tidal radius ($r_{\mathrm{t}} = 132.1$ pc). If requiring the close meet distance smaller than 60 pc (like J0731+3717), only 3 mock stars are left. The result indicates that the high-velocity halo stars in our searching volume coincidently insect with M15 by a pure chance of 1.2×10^{-5} (P_{orbit}). By an empirical cut on V_ϕ–[Fe/H] diagram (Figure S3), a total of 97,464 halo stars within the 5 kpc

searching volume are found from the existing large-scale spectroscopic surveys: SDSS DR12, LAMOST DR8, APOGEE DR16, and GALAH DR2. This means only 0.02 star could encounter with M15 within its tidal radius; but we indeed find one: the star J0731+3717.

Second, we estimate the possibility that stars possess chemical fingerprints similar to that of M15. To do so, a total of 1,930,135 stars (58,594 halo stars based on the aforementioned cut on V_ϕ–[Fe/H] diagram) with reliable determinations of [Fe/H] and [α/Fe] (requiring spectral SNR greater than 30) are selected from the existing large-scale spectroscopic surveys. Similar to the selection of high-velocity stars in ref.[11], all stars are within a (5 kpc)^3 searching volume by requiring parallax greater than 0.2 mas, parallax measurement error better than 20% and RUWE smaller than 1.4. Amongst these stars, 442 stars are found with [Fe/H] and [α/Fe] close to these of M15 within two times observational uncertainties of the cluster (see magenta box at Figure 2b). The values of [Fe/H] and [α/Fe] and their uncertainties of M15 are listed in Table 1. Therefore, 0.75% halo stars (442/58,594; $P_{\rm chem}$) in our searching volume have similar chemical pattern on [Fe/H]–[α/Fe] plane as these of M15.

Finally, we check the age differences for those halo stars with chemical pattern similar to that of M15. The distances from *Gaia* DR3 parallax measurements[11] and SDSS gri photometric observations[6] are cross-matched to those 442 aforementioned stars. By requiring photometric uncertainties smaller than 0.05 mag, 379 stars are left and shown in Figure 2c (grey diamonds). 62.8% of them (238/379; $P_{\rm CMD}$) fall into the region within the two dashed magenta lines in Figure 2c and thus they have similar age as that of M15.

Overall, there is only 5.89×10^{-8} ($P_{\rm orbit} \times P_{\rm chem} \times P_{\rm CMD}$) chance that J0731+3717 is not physically linked to M15. We thus conclude that J0731+3717 is ejected from M15 at a confidence level of "seven nines".

I Black hole mass of M15.

By applying Hills mechanism to globular cluster, the most probable ejection velocity for a star is (ref.[41]),

$$v_{\rm ej} \approx 460 \left(\frac{a}{0.1 {\rm AU}}\right)^{-1/2} \left(\frac{m}{2M_\odot}\right)^{1/3} \left(\frac{M}{10^3 M_\odot}\right)^{1/6} {\rm km\,s^{-1}}, \tag{S7}$$

where a is binary semimajor axis, m is the total mass of the binary and M is the mass of the massive black hole in the globular cluster. According to the momentum conservation, the ejection speeds for the primary and secondary are,

$$v_1 = v_{\rm ej} \left(\frac{2m_2}{m}\right)^{1/2}, \text{and } v_2 = v_{\rm ej} \left(\frac{2m_1}{m}\right)^{1/2}, \tag{S8}$$

respectively. m_1 and m_2 are the mass of primary and secondary. Following ref.[42], the possibility of an ejection is expressed as,

$$P_{\rm ej} = 1 - D/175, \tag{S9}$$

where D is a dimensionless quantity,

$$D = \left(\frac{r_{\rm close}}{a}\right) \left[\frac{2M}{10^6(m_1+m_2)}\right]^{-1/3}. \tag{S10}$$

Here $r_{\rm close}$ represents the closest distance that the binary can approach to the black hole of the cluster. $P_{\rm ej} \equiv 0$ when $D > 175$.

As shown in the Fig. S2, the maximum mass of 'undead' star in the old cluster M15 is no greater than 1 $M_\odot$. Even considering white dwarf with a mass close to Chandrasekhar limit as the companion, the mass ratio of the progenitor binary of J0731+3717 is no greater than 2. The mass ratio q is then assumed to uniformly distribute from 0 to 2. The mass of m_1 is equal to qm_2, where m_2 denotes the mass of J0731+3717. For the binary semimajor axis, we adopt the present-day distribution (with age up to 13.6 Gyr; `https://cmc.ciera.northwestern.edu/home/`) for main-sequence binaries from the Cluster Monte Carlo (CMC) N-body simulations[43]. Amongst the 148 independent N-body simulations, the model `N16-RV0.5-RG8-Z0.1` was chosen since its present-day properties (e.g., a central velocity dispersion of 9.25 km s^{-1} and a half-mass radius of 3.9 pc at 13.6 Gyr) are very similar to these of M15 (a central velocity dispersion of 13.1 km s^{-1} and a half-mass radius of 3.66 pc; ref.[44], the last update can also be found at `https://people.smp.uq.edu.au/HolgerBaumgardt/globular/parameter.html`). As same as ref.[41], $r_{\rm close}$ is assumed to uniformly distribute between 0.1 and 700 AU. Finally, considering above assumptions and the observational constraints from the ejection velocity and stellar mass, as well as their uncertainties, for J0731+3717, we generate over 200 million progenitor binaries in the MC simulations to derive the mass distribution function of the black hole. By requiring $P_{\rm ej} > 0$ and BH mass smaller than 10,000 $M_\odot$ (3σ upper limit of the central dynamic mass within M15 found by previous studies[45,46]), we find that the black hole mass of M15 is greater than 100 $M_\odot$ with a credibility of 97.784%. Moreover, the closest distance $r_{\rm close}$ between the binary and black hole is largely (93.017%) within 2 AU, as the ejection probability decreases rapidly with increasing $r_{\rm close}$ (See Fig 3 and Fig. S4 for comparison). Our comprehensive analysis strongly suggests that J0731+3717 is ejected by an IMBH hosted by M15.

Finally, we remark that we can not fully rule out close encounters happened in some rare systems, for example, a single star scattered by binary IMBHs[47]. These systems are of particular interest for various astrophysical studies but obviously far beyond the current knowledge.

J Estimate the number of J0731+3717 like star in the searching volume of current and future surveys.

The current searching volume is limited by the *Gaia* DR3 parallax measurement that is only accurate for estimating stars with distance smaller than 5 kpc (ref.[11]). For future *Gaia* DR5, the volume will be significantly expanded with distance as far as 10 kpc. We here attempt to estimate the number of J0731+3717-like stars ejected from globular cluster via Hills mechanism in current and future searching volume. Doing so, we calculate mean ejection rate for 145 well known globular clusters in the full loss-cone and empty loss-cone regimes[48]:

$$\mathcal{R}_{\rm full} = f_{\rm b}\left(\frac{a}{0.1\rm AU}\right)\left(\frac{n}{10^5\rm pc^3}\right)\left(\frac{M}{10^3 M_\odot}\right)^{4/3}\rm Myr^{-1}, \tag{S11}$$

and

$$\mathcal{R}_{\rm empty} = f_{\rm b}\left(\frac{n}{10^5\rm pc^{-3}}\right)^2\left(\frac{M}{10^3 M_\odot}\right)^3\left(\frac{\sigma}{10\rm kms^{-1}}\right)^{-9}\rm Myr^{-1}. \tag{S12}$$

Here, we set cluster binary fraction $f_{\rm b} = 8.8\%$ (ref.[49]), and a constant binary separation of 0.05 AU. The central stellar number density n is estimated by the cluster central mass density and typical mass of a star in globular cluster. The former can be found at `https://people.smp.uq.edu.au/HolgerBaumgardt/globular/parameter.html` and the latter is assumed to be $0.8\,M_\odot$. M is the mass of central black hole which is extrapolated from M-σ relation[50]. σ is the cluster central velocity dispersion that can again be found at `https://people.smp.uq.edu.au/HolgerBaumgardt/globular/parameter.html`. The number of recent ejections for each cluster can thus be calculated by $N_{\rm ej} = \overline{\mathcal{R}}\Delta T$ in the past 250 Myr (i.e. $\Delta T = 250\,\rm Myr$). $\overline{\mathcal{R}}$ is the mean of $\mathcal{R}_{\rm full}$ and $\mathcal{R}_{\rm empty}$. On average, the ejection rate is $1.34\,\rm Myr^{-1}$ for a cluster. We then run MC simulations to perform the ejection experiments. In each simulation, all the orbits of 145 globular clusters are integrated back to 250 Myr with a time resolution of 0.1 Myr, $N_{\rm ej}$ ejection events for each cluster are thus uniformly distributed in past 250 Myr. For each event, a star is assumed to be kicked off from the cluster isotropically by a velocity $v_{\rm ej}$, which can be calculated from Equations 7 and 8 by assuming a binary containing two J0731+3717 like stars with a constant separation of 0.05 AU. All these ejected stars are then integrated to present-day. In total, we performed 100 MC simulations. On average, over thirty thousand J0731+3717 like high-velocity stars with $V_{\rm GSR} \geq 400\,\rm km\,s^{-1}$ are kicked off from clusters, corresponding to an ejection rate of around $10^{-4}\,\rm yr^{-1}$, which is well consistent with that estimated from a dynamical numerical simulation (ref.[52]). As a comparison, only 10 such high-velocity stars, ejected in the past 250 Myr are found in the Monte Carlo *N*-body simulations (`https://zenodo.org/record/7599871`) for single-binary interaction involving compact objects by ref.[51]. Its rate, about $4\times10^{-8}\,\rm yr^{-1}$, is three orders of magnitude lower than that of Hills mechanism we consider here. In our simulations, the number of J0731+3717 like stars within 5 and 10 kpc from the Sun are found to be 49^{+7}_{-7} and 480^{+48}_{-32}, respectively. The values and uncertainties are given by the PDF generated by 100 MC simulations. If we consider the past 14 Gyr (the age of universe) instead of the above 250 Myr, the number of ejected stars within 5 kpc from the Sun is 537^{+24}_{-27} (without considerations of dynamical effects). This result shows that the current searching volume potentially contains a large number of high-velocity stars ejected from globular clusters but their orbits are difficult to be linked to their host clusters since most of them are not ejected recently. We note that all the above simulation results are based on a series of parameters from previous studies, which may still be under debate. However, the predicted order of magnitude is meaningful.

Currently, we have found one of the nearly hundred clusters ejected high-velocity stars as predicted by our above MC simulations. We expect dozens of more such star(s) can be discovered in the near future, with the fast increasing sampling rate of large-scale spectroscopic surveys (e.g., the LAMOST, SDSS-V and DESI). With the final release of *Gaia* DR5, the number can be even increased to a factor of ten, more details about the IMBHs hosted by globular clusters can be explored at that time.

Reference

1. GRAVITY Collaboration, Abuter, R., Amorim, A., et al. 2019, Astron. Astrophys., 625, L10. doi:10.1051/0004-6361/201935656
2. Bland-Hawthorn, J. & Gerhard, O. 2016, ARA&A, 54, 529. doi:10.1146/annurev-astro-081915-023441
3. Bovy, J. 2015, Astrophys. J. Supp., 216, 29. doi:10.1088/0067-0049/216/2/29
4. Huang, Y., Liu, X.-W., Yuan, H.-B., et al. 2015, Mon. Not. R. Astron. Soc., 449, 162. doi:10.1093/mnras/stv204
5. Kunder, A., Kordopatis, G., Steinmetz, M., et al. 2017, Astron. J., 153, 75. doi:10.3847/1538-3881/153/2/75
6. Alam, S., Albareti, F. D., Allende Prieto, C., et al. 2015, Astrophys. J. Supp., 219, 12. doi:10.1088/0067-0049/219/1/12
7. Luo, A.-L., Zhao, Y.-H., Zhao, G., et al. 2015, Research in Astronomy and Astrophysics, 15, 1095. doi:10.1088/1674-4527/15/8/002
8. Jönsson, H., Holtzman, J. A., Allende Prieto, C., et al. 2020, Astron. J., 160, 120. doi:10.3847/1538-3881/aba592
9. Buder, S., Asplund, M., Duong, L., et al. 2018, Mon. Not. R. Astron. Soc., 478, 4513. doi:10.1093/mnras/sty1281
10. Gaia Collaboration, Vallenari, A., Brown, A. G. A., et al. 2023, Astron. Astrophys., 674, A1. doi:10.1051/0004-6361/202243940
11. Li, Q.-Z., Huang, Y., Dong, X.-B., et al. 2023, Astron. J., 166, 12. doi:10.3847/1538-3881/acd1dc
12. Xiang, M. S., Liu, X. W., Yuan, H. B., et al. 2015, Mon. Not. R. Astron. Soc., 448, 822. doi:10.1093/mnras/stu2692
13. Price-Whelan, A. M. 2017, The Journal of Open Source Software, 2, 388. doi:10.21105/joss.00388
14. Chandrasekhar, S. 1943, Astrophys. J., 97, 255. doi:10.1086/144517
15. Fritz, T. K., Battaglia, G., Pawlowski, M. S., et al. 2018, Astron. Astrophys., 619, A103. doi:10.1051/0004-6361/201833343
16. Binney, J. & Tremaine, S. 2008, Galactic Dynamics: Second Edition, by James Binney and Scott Tremaine. ISBN 978-0-691-13026-2 (HB). Published by Princeton University Press, Princeton, NJ USA, 2008.

17. Bailer-Jones, C. A. L., Rybizki, J., Andrae, R., et al. 2018, Astron. Astrophys., 616, A37. doi:10.1051/0004-6361/201833456
18. Schönrich, R., Binney, J., & Dehnen, W. 2010, Mon. Not. R. Astron. Soc., 403, 1829. doi:10.1111/j.1365-2966.2010.16253.x
19. Zhou, Y., Li, X., Huang, Y., et al. 2023, Astrophys. J., 946, 73. doi:10.3847/1538-4357/acadd9
20. Reid, M. J., Menten, K. M., Brunthaler, A., et al. 2014, Astrophys. J., 783, 130. doi:10.1088/0004-637X/783/2/130
21. Harris, W. E. 2010, arXiv:1012.3224. doi:10.48550/arXiv.1012.3224
22. Vasiliev, E. & Baumgardt, H. 2021, Mon. Not. R. Astron. Soc., 505, 5978. doi:10.1093/mnras/stab1475
23. Baumgardt, H. & Vasiliev, E. 2021, Mon. Not. R. Astron. Soc., 505, 5957. doi:10.1093/mnras/stab1474
24. Erkal, D., Belokurov, V., Laporte, C. F. P., et al. 2019, Mon. Not. R. Astron. Soc., 487, 2685. doi:10.1093/mnras/stz1371
25. Patel, E., Kallivayalil, N., Garavito-Camargo, N., et al. 2020, Astrophys. J., 893, 121. doi:10.3847/1538-4357/ab7b75
26. Yanny, B., Rockosi, C., Newberg, H. J., et al. 2009, Astron. J., 137, 4377. doi:10.1088/0004-6256/137/5/4377
27. Lee, Y. S., Beers, T. C., Sivarani, T., et al. 2008, Astron. J., 136, 2022. doi:10.1088/0004-6256/136/5/2022
28. Lee, Y. S., Beers, T. C., Sivarani, T., et al. 2008, Astron. J., 136, 2050. doi:10.1088/0004-6256/136/5/2050
29. Husser, T.-O., Wende-von Berg, S., Dreizler, S., et al. 2013, Astron. Astrophys., 553, A6. doi:10.1051/0004-6361/201219058
30. Prugniel, P. & Soubiran, C. 2001, Astron. Astrophys., 369, 1048. doi:10.1051/0004-6361:20010163
31. Adelman-McCarthy, J. K., Agüeros, M. A., Allam, S. S., et al. 2008, Astrophys. J. Supp., 175, 297. doi:10.1086/524984
32. Salpeter, E. E. 1955, Astrophys. J., 121, 161. doi:10.1086/145971
33. Dotter, A., Chaboyer, B., Jevremović, D., et al. 2008, Astrophys. J. Supp., 178, 89. doi:10.1086/589654
34. Schlegel, D. J., Finkbeiner, D. P., & Davis, M. 1998, Astrophys. J., 500, 525. doi:10.1086/305772
35. Yuan, H. B., Liu, X. W., & Xiang, M. S. 2013, Mon. Not. R. Astron. Soc., 430, 2188. doi:10.1093/mnras/stt039
36. An, D., Johnson, J. A., Clem, J. L., et al. 2008, Astrophys. J. Supp., 179, 326. doi:10.1086/592090
37. Stetson, P. B. 1987, PASP, 99, 191. doi:10.1086/131977
38. Stetson, P. B. 1994, PASP, 106, 250. doi:10.1086/133378
39. Sandage, A., Katem, B., & Sandage, M. 1981, Astrophys. J. Supp., 46, 41, doi:10.1086/190734
40. Anguiano, B., Majewski, S. R., Hayes, C. R., et al. 2020, Astron. J., 160, 43. doi:10.3847/1538-3881/ab9813
41. Bromley, B. C., Kenyon, S. J., Geller, M. J., et al. 2006, Astrophys. J., 653, 1194. doi:10.1086/508419
42. Hills, J. G. 1988, Nature, 331, 687. doi:10.1038/331687a0
43. Kremer, K., Ye, C. S., Rui, N. Z., et al. 2020, Astrophys. J. Supp., 247, 48. doi:10.3847/1538-4365/ab7919
44. Baumgardt, H. & Hilker, M. 2018, Mon. Not. R. Astron. Soc., 478, 1520. doi:10.1093/mnras/sty1057
45. Gerssen, J., van der Marel, R. P., Gebhardt, K., et al. 2002, Astron. J., 124, 3270. doi:10.1086/344584
46. Gerssen, J., van der Marel, R. P., Gebhardt, K., et al. 2003, Astron. J., 125. doi:10.1086/345574
47. Fragione, G. & Bromberg, O. 2019, Mon. Not. R. Astron. Soc., 488, 4370. doi:10.1093/mnras/stz2024
48. Šubr, L., Fragione, G., & Dabringhausen, J. 2019, Mon. Not. R. Astron. Soc., 484, 2974. doi:10.1093/mnras/stz162
49. Ji, J. & Bregman, J. N. 2015, Astrophys. J., 807, 32. doi:10.1088/0004-637X/807/1/32
50. Gültekin, K., Richstone, D. O., Gebhardt, K., et al. 2009, Astrophys. J., 698, 198. doi:10.1088/0004-637X/698/1/198
51. Cabrera, T. & Rodriguez, C. L. 2023, Astrophys. J., 953, 19. doi:10.3847/1538-4357/acdc22
52. Fragione, G. & Gualandris, A. 2019, Mon. Not. R. Astron. Soc., 489, 4543. doi:10.1093/mnras/stz2451

Table S1: Top 5 globular clusters sorted by the ratio of the closest orbital distance to the clusters' tidal radius (from small to large) in the backward orbital analysis of J0731+3717.

Cluster name	Closest distance ($d_{\min}$) (kpc)	Backward time (Myr)	Tidal radius (r_t) (kpc)	$d_{\min}/r_{\rm t}$
M15	0.058	21.1	0.132	0.44
Pal 10	2.779	16.3	0.063	43.85
NGC 6715	12.867	46.9	0.279	46.09
NGC 7089	5.813	17.5	0.111	52.47
NGC 6121	2.845	1.7	0.054	52.74

Table S2: Backward orbital results under different assumptions.

Changed assumptions	Closest orbital distance (pc)	Backward time (Myr)
`BovyMWPotential2014`	56.3	21.3
$(U_\odot, V_\odot, W_\odot) = (11.10, 12.24, 7.25)\,\mathrm{km\,s^{-1}}$	57.8	21.1
$V_c(R_0) = 234.04\,\mathrm{km\,s^{-1}}$	59.1	21.1
$R_0 = 8.34\,\mathrm{kpc}$	53.1	21.1
Modelling M15 as a moving Plummer potential	55.6	21.1
Adding potential from LMC	50.9	21.1

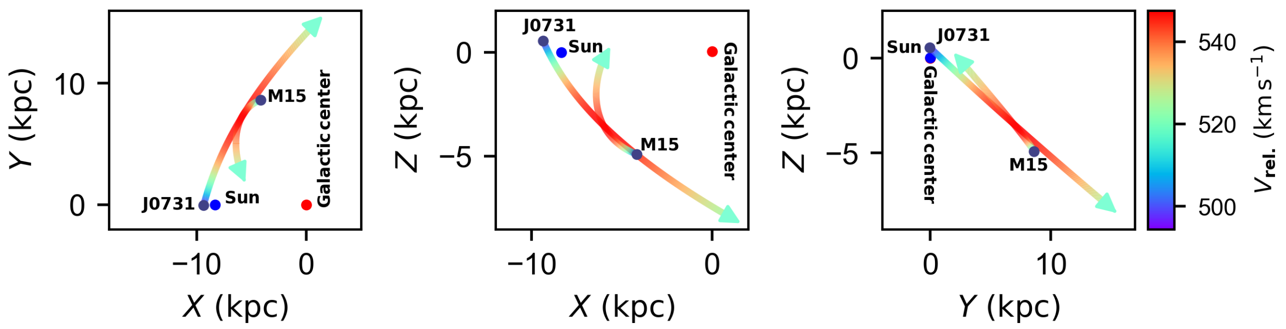

Figure S1: The 3D backward orbits of J0731+3717 and M15 projected in X–Y (left), X–Z (middle) and Y –Z (right) planes, color coded by the relative velocity between each other as indicated by the right colorbar. The positions of the Galactic center and the Sun are represented by the red and blue dots, respectively.

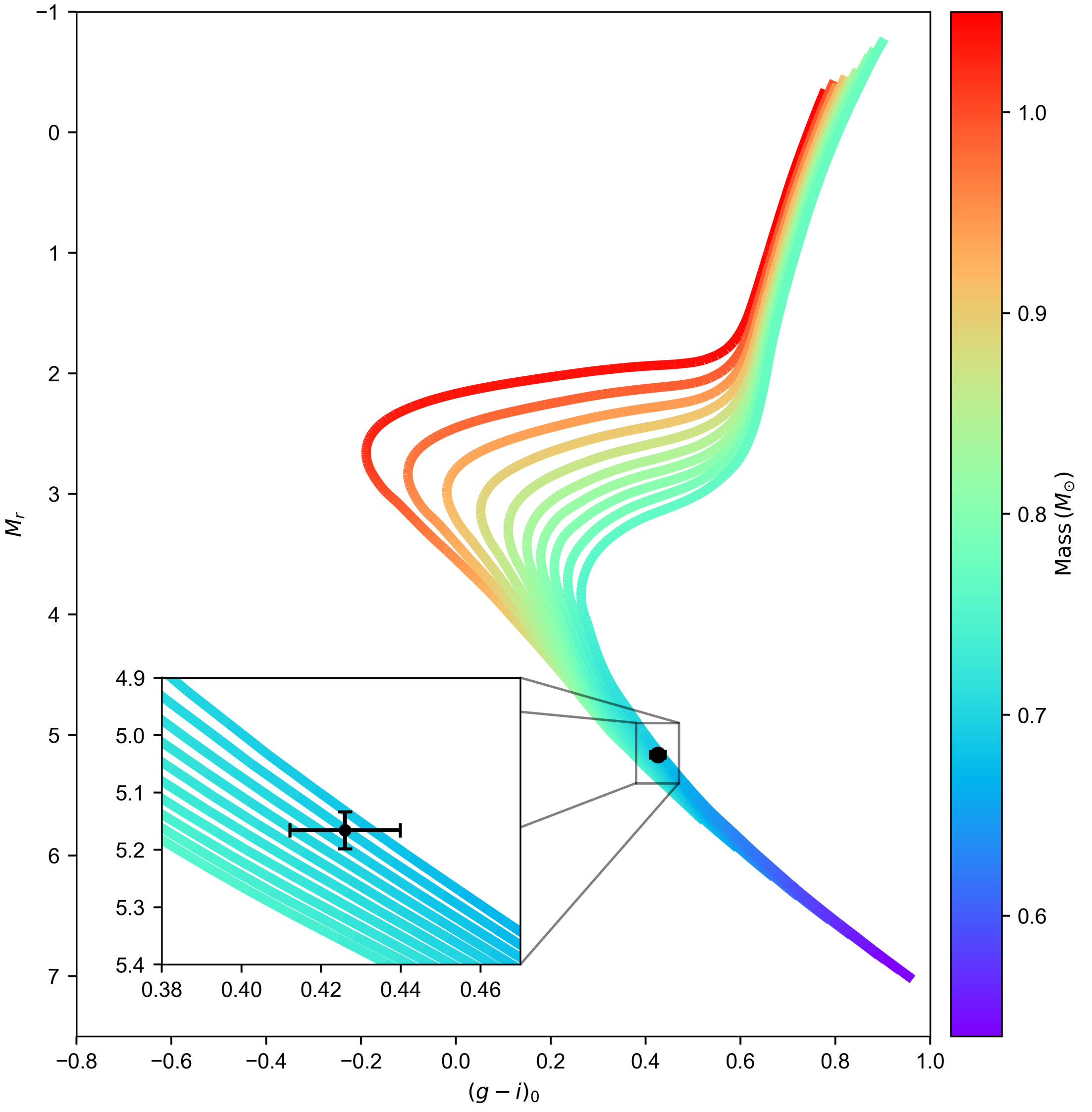

Figure S2: Comparison of J0731+3717 and stellar isochrones on $(g - i)_0$ versus M_r diagram. The black dot with error bar marks the position of J0731+3717. The background shows ten isochrones with ages ranging from 5 to 14 Gyr in a step of 1 Gyr (left to right) taken from DSEP. The colors represent the stellar mass, as indicated by the right color bar. All the isochrones have constant [Fe/H] of -2.23 and [α/Fe] of $+0.20$. Zoom inset shows the comparison more clearly.

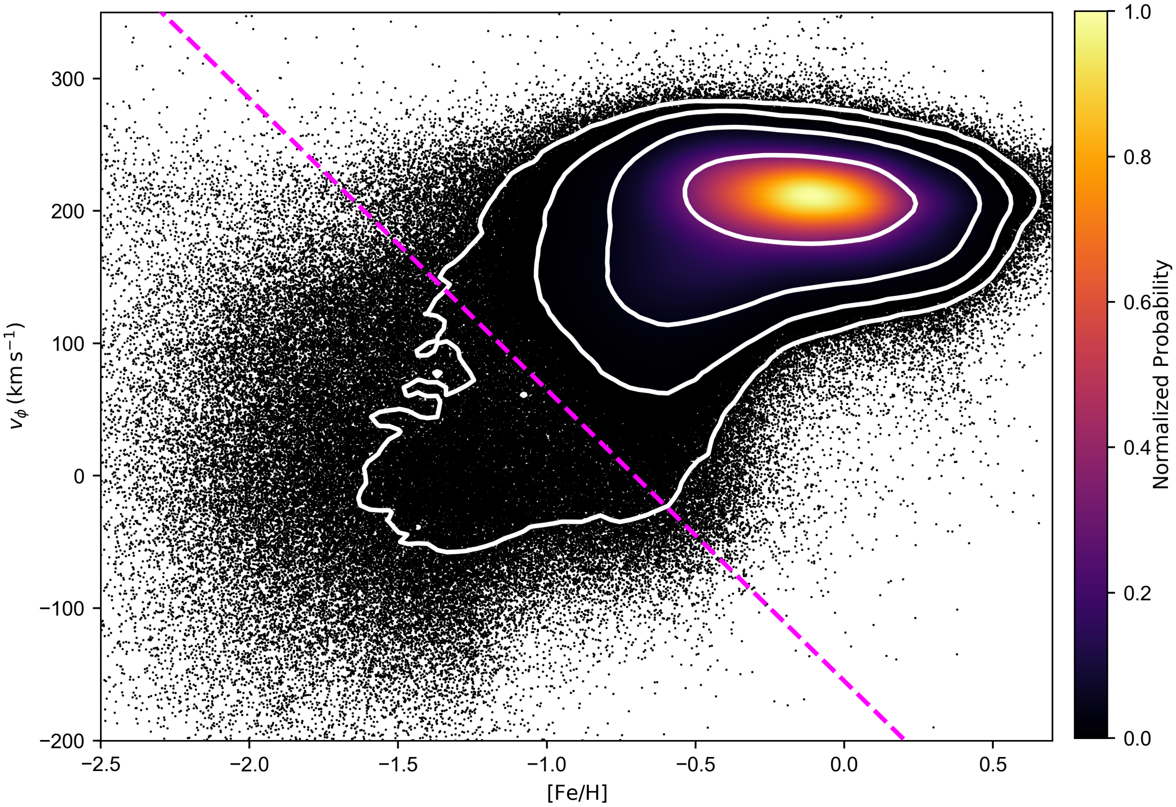

Figure S3: The density distribution of v_ϕ vs. [Fe/H] for 5,020,788 stars observed by SDSS DR12, LAMOST DR8, APOGEE DR16, and GALAH DR2 with spectral signal-to-noise ratio greater than 10. Similar to ref.[11], all stars are required to have parallax greater than 0.2 mas, parallax measurement uncertainty better than 20% and RUWE smaller than 1.4. The magenta dashed-line is an empirical cut to separate the disk population (upper right) and halo population (lower left).

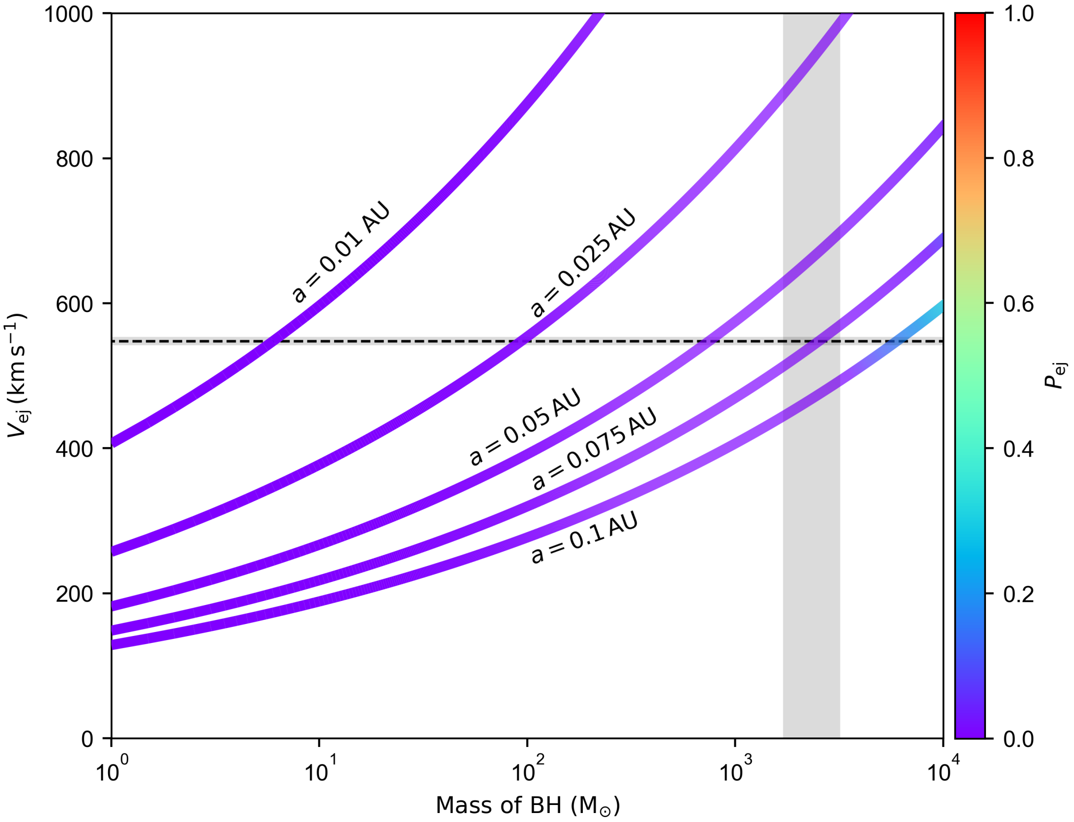

Figure S4: Ejection velocities predicted by Hills mechanism. This plot resembles Fig. 3, but with the closest distance between the binary and the black hole set to 3 AU. The ejection probability, as indicated by the colorbar on the right side, is almost zero for most black hole masses and binary separation configurations.
